# The Iowa Idea

**Published:** 1913-04

**Authors:** 


**"The Iowa Idea."**

We have before us an article on WOMEN. This is the third of a series, and the thematic substance of this one is *Building a Better Race.*

The writer, Mabel Potter Daggett, in *"The World's Work,"* tells most interestingly of a few of the practical methods now being tried out in the "Building of a Better Race."

The women of Iowa have originated what they call the "Iowa Idea," which is a plan to make Eugenics practical.

His majesty, the baby, is one of the prize products of the State Fair—not the baby with the prettiest dimples and pinkest cheeks, as formerly, but the baby whose height, weight, measurements, chest circumference, quality of skin, muscle, bone structure, size of head, formation of features, disposition, energy, facial expression and attention are most nearly ideal is the one who receives the prize.

The official score card calls for information as to parentage, environment and feeding, etc. The mothers are shown wherein the babies do not measure up to the standard. They are invited to attend the lectures, given throughout fair week, by women physicians, on hygiene, diabetes and psychological training. The writer declares, "When the present haphazard methods in this respect have been superseded, it is expected that social ostracism will be meted out to fathers and mothers who bring into the world any other kind."

The writer further declares that the women "setting out to check the great white plague came upon the *greater* black plague, and were simply appalled to discover that it was the cause of so much of the blindness, paralysis, idiocy, insanity and other troubles that have for generations been called the 'visitations of Providence.'" One of the most hopeful signs of the day is the fact that the women themselves have united to break this "conspiracy of silence."

Another means of upbuilding the race is the demand for a law that shall require, with the marriage certificate, a clean bill of health for those about to be united in marriage. And yet, declares the writer, "When the scientists shall have contributed of all their knowledge, the task will really await at the foundation which is the perfect parent."

The writer also calls our attention to the dreadful fact that 13,000 women die annually from diseases incident to childbirth. In order to overcome this evil Iowa and New York are providing county hospitals where the mothers can be cared for during parturition and confinement or any other illness that calls for skilled medical treatment. Then, too, the feeling is growing that motherlove is not all that is needed in the rearing of babies. It seems so good to know that in Chicago during the last year, "Little Mother classes" were established in thirty-one public schools and that a thousand girls between the age of 12 and 15 are being instructed in the care of babies. Philadelphia has established a similar addition to its public school curriculum, with a real live baby provided for the model in each class. The girls are taught to bathe, dress and feed this baby.

What are we doing along these lines in Texas? Only a few days ago a bill was introduced in the State Senate to prohibit the breeding of the lunatic, the criminal and the pauper. Did it pass? Indeed no! Some of those brilliant(?) Senators lifted their eloquent voices in protest, declaring that we were putting humanity *down* on a level with cattle. It is almost laughable. The real trouble is, we have never *placed humanity up* on a level with our live stock. The average farmer takes a great deal more care in the selection of a sire for his mare than he does in the choice of a husband for his daughter, a father for his grandchildren.

Shall not we, the women of Texas, inform ourselves on these subjects, and discuss them in our homes and with our friends until we create public sentiment in favor of the Science of Eugenics and Home Economics?

